# Presynaptic G Protein-Coupled Receptors: Gatekeepers of Addiction?

**DOI:** 10.3389/fncel.2016.00264

**Published:** 2016-11-11

**Authors:** Kari A. Johnson, David M. Lovinger

**Affiliations:** Section on Synaptic Pharmacology, Laboratory for Integrative Neuroscience, National Institute on Alcohol Abuse and Alcoholism, National Institutes of HealthBethesda, MD, USA

**Keywords:** addiction, self-administration, presynaptic, GPCR, dopamine receptor, CB1 receptor, metabotropic glutamate receptor, allosteric modulator

## Abstract

Drug abuse and addiction cause widespread social and public health problems, and the neurobiology underlying drug actions and drug use and abuse is an area of intensive research. Drugs of abuse alter synaptic transmission, and these actions contribute to acute intoxication as well as the chronic effects of abused substances. Transmission at most mammalian synapses involves neurotransmitter activation of two receptor subtypes, ligand-gated ion channels that mediate fast synaptic responses and G protein-coupled receptors (GPCRs) that have slower neuromodulatory actions. The GPCRs represent a large proportion of neurotransmitter receptors involved in almost all facets of nervous system function. In addition, these receptors are targets for many pharmacotherapeutic agents. Drugs of abuse directly or indirectly affect neuromodulation mediated by GPCRs, with important consequences for intoxication, drug taking and responses to prolonged drug exposure, withdrawal and addiction. Among the GPCRs are several subtypes involved in presynaptic inhibition, most of which are coupled to the G_i/o_ class of G protein. There is increasing evidence that these presynaptic G_i/o_-coupled GPCRs have important roles in the actions of drugs of abuse, as well as behaviors related to these drugs. This topic will be reviewed, with particular emphasis on receptors for three neurotransmitters, Dopamine (DA; D_1_- and D_2_-like receptors), Endocannabinoids (eCBs; CB1 receptors) and glutamate (group II metabotropic glutamate (mGlu) receptors). The focus is on recent evidence from laboratory animal models (and some evidence in humans) implicating these receptors in the acute and chronic effects of numerous abused drugs, as well as in the control of drug seeking and taking. The ability of drugs targeting these receptors to modify drug seeking behavior has raised the possibility of using compounds targeting these receptors for addiction pharmacotherapy. This topic is also discussed, with emphasis on development of mGlu_2_ positive allosteric modulators (PAMs).

## Introduction

Drug and alcohol use disorders are prominent neuropsychiatric conditions that create substantial economic, health and societal costs. Substance abuse definitions have evolved over time, but key features include relapse to drug use even after prolonged abstinence, escalation of drug intake, tolerance to the effects of drugs, craving, and continued use despite adverse consequences to health, financial status and relationships (American Psychiatric Association, 2013; Hasin et al., 2013; Koob and Volkow, 2016). Repeated drug exposure produces neuroadaptations that contribute to pathological drug-related behaviors. These include long-term alterations in gene expression, protein regulation, anatomy, and synaptic function that collectively influence neural circuits that govern reward, motivation, and action control to produce maladaptive behaviors. In particular, abused drugs produce adaptations in limbic, associative, and sensorimotor cortico-basal ganglia circuits that alter responses to drugs and contribute to inflexible drug taking and seeking behaviors (for review see Gremel and Lovinger, 2016; Scofield et al., 2016). Drug-associated changes in the activity of the dorsal and ventral striatum (nucleus accumbens, NAc) heavily contribute to various aspects of drug intake. In addition, inputs to the NAc from regions such as the amygdala and bed nucleus of the stria terminalis (BNST) provide information about environmental stimuli associated with drug intake that contributes to relapse to drug seeking following abstinence (Stamatakis et al., 2014). Plasticity of glutamatergic neurotransmission in these circuits has been implicated as a major neurobiological process underlying addictive behaviors (Kalivas, 2009; Scofield et al., 2016). Therefore, it is critical to understand the cellular processes that contribute to drug-induced plasticity. Moreover, a reversal of synaptic dysfunction in these circuits could correct pathological behaviors and represents a promising approach to designing new therapeutic strategies for drug use disorders. In this context, neurotransmitter receptors that are poised to influence synaptic transmission in key addiction-relevant circuits are likely to play critical roles in the effects of abused drugs and as novel therapeutic targets.

**Figure 1:**
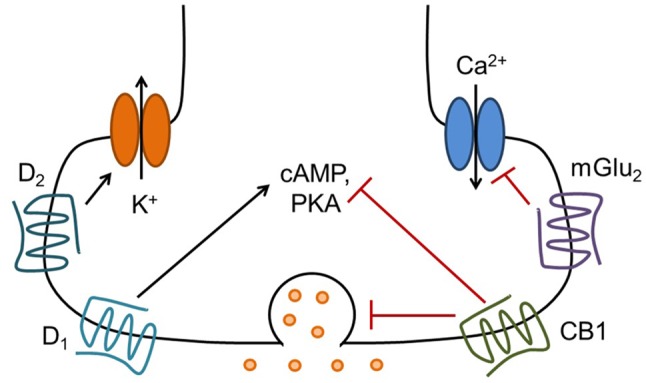
**Presynaptic G protein-coupled receptors (GPCRs) modulate neurotransmitter release via several mechanisms.** Presynaptic G_i/o_-coupled GPCRs such as mGlu_2_, cannabinoid type 1 (CB1) and D_2_ can reduce the probability of neurotransmitter release by inhibiting calcium influx through voltage-gated calcium channels, by directly modulating the function of vesicle release machinery, and possibly by activating inwardly rectifying potassium channels to hyperpolarize or shunt the presynaptic terminal. These receptors also reduce cyclic adenosine monophosphate (cAMP) levels and protein kinase A (PKA) activity via inhibition of adenylyl cyclase, which may contribute to long-term regulation of neurotransmitter release. Conversely, presynaptic D_1_ receptors, which are coupled to G_s_/G_olf_ and activate PKA signaling, can increase neurotransmitter release. There are likely many other mechanisms involved in the regulation of neurotransmitter release by GPCRs, including activation of other signaling pathways and stimulation of protein synthesis (see Atwood et al., 2014b for further discussion).

G protein-coupled receptors (GPCRs; also known as 7 transmembrane domain or 7TM receptors) are a large class of metabotropic receptors for neurotransmitters and hormones that couple to heterotrimeric G proteins. G proteins mediate a wide variety of cellular functions, including, but not limited to, altering the production of second messengers such as cyclic adenosine monophosphate (cAMP), mobilizing internal calcium stores, modulating ion channel function, altering neurotransmitter release, and influencing gene expression. Upon activation of a GPCR, the heterotrimeric G protein dissociates into α and βγ subunits, which then modulate the function of a diverse array of effector proteins to simultaneously influence many cellular functions (Latek et al., 2012). GPCRs are often classified by the G protein α subunits with which they prefer to interact. This review will primarily focus on Gα_i/o_-coupled GPCRs, which are well-known modulators of neurotransmitter release (Figure 1). These receptors inhibit adenylyl cyclase to reduce production of the second messenger molecule cAMP. In addition to Gα-mediated effects, the Gβγ subunits liberated by GPCR activation can influence neuronal physiology via direct interactions with voltage-gated calcium channels, G protein-activated inward-rectifying potassium (GIRK) channels, and vesicular release machinery. In the context of the presynaptic axon terminal, each of these mechanisms can contribute to the inhibition of neurotransmitter release in response to GPCR activation (Kretz et al., 1986; Herlitze et al., 1996; Ikeda, 1996; Seino and Shibasaki, 2005).

Preclinical models of drug abuse including investigator-administered passive drug exposure, conditioned place preference (CPP) and operant drug self-administration have been used to evaluate the multifaceted relationships between presynaptic GPCRs and drugs of abuse (for reviews of common animal models, see Belin-Rauscent et al., 2016; Scofield et al., 2016). Based on these studies, several prominent themes have emerged. First, GPCRs, particularly Dopamine (DA) receptors, are important mediators of the neurochemical and behavioral effects of drugs of abuse. Second, modulation of presynaptic GPCRs can alter neurochemical and behavioral responses to acute drug exposure in ways that would be predicted to either promote or constrain the rewarding and stimulating effects of a variety of abused drugs. Third, repeated exposure to drugs such as alcohol, cocaine, and nicotine produce long-lasting changes in the ability of some presynaptic GPCRs to modulate the release of neurotransmitters, particularly glutamate, in addiction-relevant circuits. Such drug-induced neuroadaptations are likely to play key roles in the transitions to problematic drug-related behaviors including escalation of drug taking and relapse following abstinence. Finally, pharmacological manipulation of presynaptic GPCRs can reduce seeking and taking of self-administered drugs in both rodent and non-human primate models, suggesting that targeting these receptors could be a viable therapeutic approach for treating addiction. The present review will highlight these themes using the examples of three well-known classes of GPCRs: DA receptors, which are important mediators of the CNS effects of abused drugs; cannabinoid type 1 (CB1) receptors; and group II metabotropic glutamate receptors (mGlus). We will then discuss recent progress towards translating preclinical findings into clinical therapeutics.

## Dopamine Receptors

DA is one of the few small molecule neurotransmitters that exclusively activate GPCRs in the mammalian brain, and thus only has modulatory neurophysiological and neurochemical effects. There are five different DA receptor subtypes (D_1–5_) with the D_1_ and _−5_ subtypes generally coupled to G_s/olf_ G proteins, and the D_2_, _−3_ and _−4_ coupled to G_i/o_ (for review see Beaulieu and Gainetdinov, 2011). DA receptors are found on both pre- and postsynaptic elements of many CNS neuronal subtypes. In some neurons, such as striatal medium spiny projection neurons (MSNs), it is likely that DA receptors mediate effects that are considered postsynaptic (e.g., changes in intracellular signaling, gene expression, and modulation of postsynaptic ionotropic glutamate receptor function) as well as effects that are presynaptic (i.e., modulating neurotransmitter release onto neighboring MSNs or neurons in striatal target regions such as the globus pallidus/ventral pallidum). The anatomical distribution of dopaminergic neurons is well circumscribed within the brain. The large majority of dopaminergic neuronal somata reside within the A9 and A10 ventral midbrain areas, also known as the substantia nigra pars compacta (SNc) and ventral tegmental area (VTA), respectively. Efferent projections from these nuclei give rise to dense axonal fields in the dorsal striatum (from SNc) and ventral striatum/NAc (from VTA). Sparser DA projections innervate areas in the frontal cortex and allocortical areas such as the amygdala and hippocampus (Scatton et al., 1980; Gasbarri et al., 1997; Bjorklund and Dunnett, 2007; Lammel et al., 2008). These non-striatal projections mainly arise from the VTA (Gasbarri et al., 1997), although the SNc does give rise to a small hippocampal projection (Scatton et al., 1980). Midbrain dopaminergic neurons have crucial roles in movement initiation and control, signaling reward and salience of environmental events, and several other brain functions (Schultz, 2007; Wickens et al., 2007; Palmiter, 2008; Yin, 2016). A second, lesser known source of dopaminergic neurons is the ventral periaqueductal gray (PAG)/dorsal raphe nucleus (DRN) border region (Dougalis et al., 2012). This projection may have important roles in central processing of pain- and stress-related stimuli, but will not be discussed in detail in this review article.

Dopaminergic transmission is the direct target of a number of drugs of abuse, and is secondarily affected by virtually all abused drugs (reviewed in Volkow and Morales, 2015). The “stimulant” drugs such as cocaine, amphetamines and methylphenidate produce many of their CNS actions via molecular interactions with the dopamine transporter (DAT). This transporter is the main conduit for clearance of extracellular DA, strongly controlling effective concentrations of the neurotransmitter. Cocaine and methylphenidate inhibit transporter function, effectively blocking the reuptake of extracellular DA. The stimulant drugs thus prolong the time course and increase the extracellular spread of DA following vesicular release. Amphetamines can activate the reverse transport of intracellular DA to increase the extracellular content. This action also prevents uptake via the transporter, leading to large increases and spread of extracellular DA. The net effect of all these stimulant drug effects is to produce longer-lasting and more widespread activation of DA receptors, with subtle but important differences between the pure uptake blockers and amphetamines. The impact of these DAT-targeted drug effects are largest in the striatum, as the transporter is most highly expressed in the terminal fields in this structure. However, lesser effects can occur in cortical regions and even in the midbrain itself. Little is known about stimulant drug effects on PAG neurons and their axon terminals.

Other drugs of abuse increase dopaminergic synaptic transmission via distinct processes. One prominent mechanism is the disinhibition of midbrain dopaminergic neurons due to decreased activity or synaptic transmission from GABAergic neurons. This mechanism likely accounts for the DA enhancing effects of, benzodiazepines, cannabinoids, and opiates (Johnson and North, 1992; Szabo et al., 2002; Lupica and Riegel, 2005; Tan et al., 2010), and plays a part in the actions of nicotine (Pidoplichko et al., 2004). In the case of cannabinoids, G_i/o_-coupled GPCRs activated by these drugs are present on GABAergic presynaptic terminals that synapse onto midbrain dopaminergic neurons (Szabo et al., 2002; Lupica and Riegel, 2005). Activation of these GPCRs inhibits GABA release, effectively removing inhibition of DA neurons and allowing for greater firing and more DA release. Ethanol also appears to disinhibit dopaminergic neurons via effects on the firing of GABAergic neurons (Stobbs et al., 2004; Tateno and Robinson, 2011; but see Theile et al., 2011), although ethanol can also directly excite midbrain dopaminergic neurons via more direct effects on the neurons themselves (Melis et al., 2009; Morikawa and Morrisett, 2010).

As with the stimulant drugs, the main impact of other drugs of abuse on dopaminergic transmission occurs in target regions of the dopaminergic afferents (i.e., striatum and frontal cortex). All of these drugs increase extracellular DA in the dorsal and ventral striatum (Di Chiara and Imperato, 1988; Benwell and Balfour, 1997; Mathews et al., 2006). Early reports indicated that these effects were larger in the ventral striatum, and this region has been implicated in several aspects of drug use, abuse and relapse (Di Chiara and Imperato, 1988). However, drug effects in the dorsal striatum should not be ignored, as they likely contribute to the learning of goal-directed and habitual actions related to drug seeking and continued use (Gremel and Lovinger, 2016). Despite all that is known about the increases in extracellular DA produced by drugs of abuse, less is known about which receptors are responsible for translating this increase into intoxication and other acute drug actions. This subject clearly requires considerable additional research, but is outside the focus on presynaptic receptors in the present review article.

The roles of DA receptors in the effects of stimulant drugs have been examined in electrophysiological experiments. Application of amphetamine generally increases the firing of striatal neurons (e.g., Rebec et al., 1997; Glynn and Ahmad, 2003). Cocaine and amphetamine reliably inhibit afferent excitability and synaptic transmission in ventral striatum (Garcia-Munoz et al., 1991; Harvey and Lacey, 1996; Nicola et al., 1996; Glynn and Ahmad, 2003; Adrover et al., 2014; Dobbs et al., 2016), the globus pallidus and ventral pallidum (Floran et al., 1997; Dobbs et al., 2016), and the amygdala (Huang et al., 2003). In the ventral striatum, this effect appears to involve presynaptic suppression of neurotransmitter release and D_1_ receptor activation (Harvey and Lacey, 1996; Nicola et al., 1996). Although many physiological and behavioral effects of stimulant drugs cannot currently be attributed to specific pre- or postsynaptic receptor populations, a recent report by Dobbs et al. (2016) begins to shed light on this important question. This study found that cocaine increases firing of D_1_-expressing MSNs by reducing collateral inhibition via activation of D_2_ receptors on D_2_-expressing MSNs, and that presynaptic MSN D_2_ receptors are critical mediators of acute locomotor responses to cocaine (Dobbs et al., 2016).

Changes in DA receptor expression and function have been examined following chronic exposure to both stimulant and non-stimulant drugs of abuse. For example, chronic cocaine exposure leads to D_1_ supersensitivity in NAc (Nestler and Aghajanian, 1997), and increases in D_2_ receptor antagonist binding sites in the DS and NAc (Goeders and Kuhar, 1987). Increased sensitivity to behavioral effects of D_2_ agonists have also been observed (Ujike et al., 1990). However, reduced sensitivity of presynaptic autoreceptors, DA receptors on the dopaminergic neurons themselves, has also been observed following repeated cocaine administrations (Yi and Johnson, 1990). Imaging studies in both animals and humans reveal that decreased D_2_ receptor availability is a striking common feature of chronic exposure to drugs of abuse including cocaine, alcohol, methamphetamine, heroin, nicotine, and cannabis (for review see Volkow et al., 2009). Importantly, reduced D_2_ function following long-term drug abuse is thought to underlie reduced motivation for natural reinforcers.

The extent to which DA receptor ligands can mimic or alter behavioral actions of drugs of abuse has been widely studied (Table 1). Ligands for both D_1_ and D_2_ have cocaine-like effects when given acutely, and antagonists can block acute cocaine actions (Spealman et al., 1992; Baik, 2013). Activation of D_2_ receptors has been shown to blunt cocaine sensitization (Beyer and Steketee, 2002), while D_2_ antagonists or receptor knockout generally does not greatly alter acute locomotor activation, sensitization, or CPP (Spyraki et al., 1982; Cabib et al., 1991; Kuribara and Uchihashi, 1993; Mattingly et al., 1994; Cervo and Samanin, 1995; Shippenberg and Heidbreder, 1995; Ushijima et al., 1995; Vanderschuren and Kalivas, 2000; Nazarian et al., 2004; Welter et al., 2007; Sim et al., 2013). Deleting only those D_2_ receptors expressed on dopaminergic neurons themselves increases CPP for cocaine (Bello et al., 2011), highlighting the fact that antagonists or global knockout may not have any net effect due to different physiological roles of receptors expressed by different neuronal subtypes. Substantial literature indicates that D_1_ receptor activation has crucial roles in psychostimulant-induced behaviors, including locomotor activation, sensitization, CPP and self-administration (Kalivas, 1995; Baik, 2013). Generally, inhibiting or knocking out D_1_ receptors will attenuate locomotor activation, and CPP for cocaine (Cabib et al., 1991; Ushijima et al., 1995; Hummel and Unterwald, 2002). However, cocaine sensitization is not greatly altered by D_1_ antagonists or knockout (Kuribara and Uchihashi, 1993; Mattingly et al., 1994; Steketee, 1998; White et al., 1998; Vanderschuren and Kalivas, 2000; Karlsson et al., 2008), although it is reduced by inactivation of D_1_-MSNs in NAc (Hikida et al., 2010; Chandra et al., 2013). Recent data from the Luscher laboratory provides compelling evidence that important aspects of the drug use disorder profile can be initiated by selective activation of the mesolimbic dopaminergic system, and that D_1_ receptors play a crucial role in these effects (Pascoli et al., 2015).

**Table 1:** **Behavioral effects of drugs targeting dopamine receptors**.

Pharmacological manipulation	Behavioral effect	Reference(s)
D_1_ agonist	cocaine-like effects	reviewed in Baik (2013)
D_1_ antagonist	↓ cocaine locomotor activation	Cabib et al., 1991; Ushijima et al., 1995
	↔ cocaine sensitization	Kuribara and Uchihashi, 1993; Mattingly et al., 1994; Steketee, 1998; White et al., 1998; Karlsson et al., 2008
	↓ methamphetamine sensitization	Kuribara and Uchihashi, 1993
	↕ cocaine SA	Woolverton, 1986; Britton et al., 1991; Corrigall and Coen, 1991a; Hubner and Moreton, 1991; Caine and Koob, 1994
	↓ amphetamine SA	Phillips et al., 1994; Pierre and Vezina, 1998
	↓ MDMA SA	Brennan et al., 2009
	↓ nicotine SA	Corrigall and Coen, 1991b; Kutlu et al., 2013; Hall et al., 2015
	↓ cocaine seeking	Brown et al., 2012
	↓ MDMA seeking	Schenk et al., 2011
	↓ nicotine seeking	Liu et al., 2010
	↓ heroin seeking	Shaham and Stewart, 1996
D_2_ agonist	cocaine-like effects	reviewed in Baik (2013)
	↓ cocaine sensitization	Beyer and Steketee, 2002
	↓ cocaine CPP	Hummel and Unterwald, 2002
	↑ cocaine seeking	Self et al., 1996; De Vries et al., 1999; Spealman et al., 1999; Khroyan et al., 2000; De Vries et al., 2002; Fuchs et al., 2002
D_2_ antagonist	↔ cocaine CPP	Spyraki et al., 1982; Cervo and Samanin, 1995; Nazarian et al., 2004
	↔ cocaine locomotor activation	Cabib et al., 1991; Ushijima et al., 1995
	↔ cocaine sensitization	Kuribara and Uchihashi, 1993; Mattingly et al., 1994; Shippenberg and Heidbreder, 1995; White et al., 1998
	↓ methamphetamine sensitization	Kuribara and Uchihashi, 1993
	↓ cocaine SA (mPFC)	Goeders and Smith, 1983
	↕ cocaine SA	Woolverton, 1986; Britton et al., 1991; Hubner and Moreton, 1991; Corrigall and Coen, 1991a; Caine and Koob, 1994
	↓ amphetamine SA	Phillips et al., 1994
	↑ MDMA SA	Brennan et al., 2009
	↓ nicotine SA	Corrigall and Coen, 1991b
	↓ nicotine seeking	Liu et al., 2010
	↓ heroin seeking	Shaham and Stewart, 1996

*SA, self-administration*.

Studies evaluating the effects of manipulating DA receptor function on drug self-administration have revealed critical roles for these receptors in drug taking. Notably, blockade of D_2_ receptors in the medial prefrontal cortex has been shown to attenuate cocaine self-administration (Goeders and Smith, 1983). Systemic administration of D2 antagonists can enhance MDMA self-administration (Brennan et al., 2009). However, peripheral antagonist injections do not have consistent effects, due in part to biphasic effects at different doses (Woolverton, 1986; Britton et al., 1991; Corrigall and Coen, 1991a; Hubner and Moreton, 1991; Witkin et al., 1991; Caine and Koob, 1994). The literature on D1 antagonist effects on cocaine self-administration shows similar variability (Woolverton, 1986; Britton et al., 1991; Hubner and Moreton, 1991; Vanover et al., 1991; Caine and Koob, 1994). Other factors that may have influenced these outcomes were species and strain differences, differences in self-administration protocol, and differential impact of blocking D_1_ receptors on different neuronal targets at different drug doses. Knocking out all D_1_ receptors eliminates cocaine self-administration (Caine et al., 2007). Self-administration of MDMA is also prevented by a D_1_ antagonist (Brennan et al., 2009). Intra-accumbens D_1_ or D_2_ antagonist injection reduces amphetamine and nicotine self-administration (Corrigall and Coen, 1991b; Phillips et al., 1994), and D_1_ antagonists also reduce drug-induced facilitation of amphetamine self-administration (Pierre and Vezina, 1998). In addition to the NAc shell subregion, the insular and parietal association cortices appear to be brain regions where D_1_ receptors control self-administration (Kutlu et al., 2013; Hall et al., 2015).

DA receptors are also involved in the reinstatement of drug seeking following abstinence. For example, cue-induced reinstatement of nicotine seeking is reduced by both D_1_ and D_2_ antagonists (Liu et al., 2010), and reinstatement of MDMA seeking is prevented by D_1_ antagonists (Schenk et al., 2011). Stress-induced reinstatement of heroin seeking is partially reduced by both D_1_ and D_2_ antagonists, with a larger effect of a non-selective DA receptor antagonist (Shaham and Stewart, 1996), whereas stress-induced reinstatement of cocaine seeking is only prevented by a D_1_ antagonist (Brown et al., 2012). Conversely, activation of D_2_ enhanced reinstatement of both cocaine and heroin seeking (De Vries et al., 1999). It has been shown in several studies that D_2_ agonists can induce reinstatement of cocaine seeking behavior (Self et al., 1996; De Vries et al., 1999, 2002; Spealman et al., 1999; Khroyan et al., 2000; Fuchs et al., 2002). Mice lacking D_2_ receptors show increased cocaine self-administration (Caine et al., 2002), but reinstatement of seeking induced by stress is attenuated in these mice (Sim et al., 2013). These seemingly conflicting results may be attributable to differences in the neurobiology of drug seeking for different drugs of abuse and different circuitry engaged by various stimuli that produce reinstatement of drug seeking.

## CB1 Receptors

Endocannabinoids (eCBs) are lipid metabolites that act as juxtacrine and paracrine modulators throughout the nervous system and body. Within the brain, eCBs produce their actions predominantly through activation of CB1, a class A GPCR that is almost exclusively localized to presynaptic terminals. The eCB name is obviously derived from the term cannabis, and this is due to the fact that CB1 is also the primary CNS molecular target for Δ^9^-tetrahydrocannabinol (Δ^9^THC), the major psychoactive ingredient in preparations of *Cannabis sativa*, which acts as a partial agonist at these receptors. The two major eCBs with synaptic actions are 2-arachidonoyl glycerol (2-AG) and arachidonoyl ethanolamide (AEA, also known as anandamide; for review see Lu and Mackie, 2016). The eCBs can be produced by virtually any cell in the brain, and the CB1 receptor shows widespread expression throughout the nervous system. The best known actions of CB1 involve presynaptic depression of neurotransmitter release, mediated via G_i/o_ activation. Downstream effectors involved in this inhibition include voltage-gated calcium channels, as-yet unidentified components of vesicle fusion machinery, and perhaps certain potassium channels (Chevaleyre et al., 2006).

A “retrograde” signaling mechanism in which eCBs are released from postsynaptic elements and act on presynaptic CB1 receptors, appears to be the predominant means for this neuromodulatory intercellular signaling. Both short- and long-term synaptic depression result from eCB signaling (Chevaleyre et al., 2006). The eCB-mediated short-term depression (STD) appears to persist only as long as eCBs occupy CB1 receptors (Heinbockel et al., 2005). The long-term depression (LTD) outlasts receptor occupancy and activation (Chevaleyre and Castillo, 2003).

Since the discovery of CB1 and eCBs in the early 1990s, literature on the actions of drugs of abuse on this system has become vast. The most obvious interactions are with Δ^9^-THC derived from phytocannabinoids. As it is a partial agonist at CB1, Δ^9^-THC can mimic the synaptic depressant effects of eCBs (Hoffman and Lupica, 2013). Synthetic CB1 agonists have also been developed (e.g., WIN 55, 212–2, HU 210, CP55, 940), and these generally have high efficacy and produce strong synaptic depressant effects. Acute treatment with Δ^9^-THC or synthetic CB1 agonists produce well-characterized signs of intoxication and anti-nociception in humans and laboratory animals through CB1 activation (Wiley and Martin, 2003). Presynaptic CB1 receptors present on GABAergic terminals in the midbrain reduce inhibition of dopaminergic neurons (for additional discussion see Wang and Lupica, 2014; Covey et al., 2015). This mechanism is thought to account for the rewarding effects of Δ^9^-THC and other cannabinoid drugs, and is also likely to be a prominent mechanism through which eCBs contribute to brain mechanisms of reward.

Chronic exposure to Δ^9^-THC produces tolerance to many of the behavioral effects seen during initial drug exposure, but it has been harder to pin down signs of withdrawal and dependence, especially in laboratory animal models (Compton et al., 1990). Humans that regularly use cannabis preparations in large doses report craving, heightened anxiety, increased irritability, decreased food intake and sleep disruption following discontinuation of use (Haney et al., 1999; Budney et al., 2001; Singleton et al., 2002; Gates et al., 2014). Other withdrawal signs are not apparent, but this is due mainly to the high lipophilicity of Δ^9^-THC which causes the drug to accumulate in fatty tissues, allowing for slow release and continuous low-level receptor activation. Following chronic Δ^9^-THC or synthetic CB1 agonist exposure, treatment with synthetic CB1 antagonists elicits signs of withdrawal including turning, chewing, digging, paw tremors and head shakes in laboratory animals (Cook et al., 1998; Rubino et al., 1998), supporting the idea these symptoms are largely suppressed by this continued post-withdrawal receptor activation. It has been hard to develop methods for self-administration of Δ^9^-THC and other CB1 agonists in laboratory animals, in part due to aversive effects of the compound. Intravenous self-administration has been reported, particularly in squirrel monkeys (Tanda et al., 2000; Justinova et al., 2003, 2005; Zangen et al., 2006), and CB1 antagonists reduce Δ^9^-THC self-administration as well as cue- and priming-induced reinstatement following extinction (Justinova et al., 2008). CPP for Δ^9^-THC and other CB1 agonists has also been demonstrated (Valjent and Maldonado, 2000; Braida et al., 2001). Notably, cannabinoid agonists can induce conditioned place aversion (CPA) in rodents, while CB1 antagonists can also induce CPP (Cheer et al., 2000). The net agonist-induced preference or aversion is critically dependent on dose. Thus, CB1 activation has mixed aversive and rewarding effects that could affect the abuse potential in different individuals. Administration of Δ^9^-THC also lowers the threshold for rewarding brain stimulation (reviewed in Gardner, 2002). Thus, there is some evidence for cannabis reward and withdrawal signs, and Δ^9^-THC clearly has abuse and dependence liability.

The eCB system is strongly implicated in the rewarding effects of a variety of abused drugs (Table 2; Justinova et al., 2009; Covey et al., 2015). Antagonists of CB1 impair acquisition or reinstatement of CPP for cocaine, morphine, and nicotine (Chaperon et al., 1998; Le Foll and Goldberg, 2004; Forget et al., 2005; Biala et al., 2009; Fang et al., 2011), but not ethanol (Pina and Cunningham, 2014). In contrast, CB1 knockout mice show reduced ethanol CPP (Houchi et al., 2005). Interestingly, CB1 knockout mice are not resistant to cocaine CPP, and in fact display a facilitation of CPP acquisition following stress (Miller et al., 2008). Consolidation, retrieval and reconsolidation of methamphetamine CPP are also disrupted by CB1 antagonists (Yu et al., 2009). The antagonist effects on morphine CPP are also observed with antagonist injection into the NAc (Azizi et al., 2009), and CB1 receptors in the central amygdala may also be involved (Rezayof et al., 2011). Nicotine CPP may also involve CB1 receptors in the basolateral amygdala (Hashemizadeh et al., 2014). ECBs and CB1 also contribute to the intake of several drugs of abuse in animal models. Both CB1 antagonist treatment and CB1 knockout decrease ethanol intake and preference in a standard two-bottle choice paradigm (reviewed in Pava and Woodward, 2012). Operant ethanol self-administration is also altered by antagonists (Freedland et al., 2001; Navarro et al., 2001; Caillé et al., 2007), and in CB1 knockout mice (Thanos et al., 2005). Considering other drugs of abuse, similar results have been obtained for self-administration of heroin (Navarro et al., 2001; Solinas et al., 2003), morphine (Cossu et al., 2001), MDMA (Sala and Braida, 2005; Touriño et al., 2008), and nicotine (Cohen et al., 2002; Shoaib, 2008). Modulation of drug-taking by CB1 receptors appears to be bidirectional, as CB1 agonist administration potentiates nicotine seeking and taking (Gamaleddin et al., 2012). Direct infusions of a CB1 antagonist into the VTA, but not the NAc, reduce nicotine self-administration (Simonnet et al., 2013), suggesting a pivotal role for VTA CB1 receptors. Different regions may be involved in reinstatement of nicotine seeking following extinction, as CB1 antagonists injected into the NAc shell, BLA, or mPFC impair reinstatement (Kodas et al., 2007). Similarly, cue-induced reinstatement of heroin seeking is inhibited by systemic injection of a CB1 antagonist or by direction infusion into the NAc core region or the PFC (Fattore et al., 2005; Caillé and Parsons, 2006; Alvarez-Jaimes et al., 2008). Systemic injections of CB1 antagonists also reduce drug-priming or cue-induced reinstatement of ethanol and nicotine seeking (Cohen et al., 2005; Forget et al., 2009; de Bruin et al., 2011). Collectively, these findings indicate that CB1 receptors in a variety of addiction-relevant brain regions contribute to various aspects of drug taking and seeking behavior, and that the receptors involved may vary between drugs of abuse.

**Table 2:** **Behavioral effects of drugs targeting cannabinoid type 1 (CB1) receptors**.

Pharmacological manipulation	Behavioral effect	Reference(s)
CB1 inverse agonist	↓ cocaine, morphine, nicotine, morphine, methamphetamine CPP	Chaperon et al., 1998; Le Foll and Goldberg, 2004; Forget et al., 2005; Azizi et al., 2009; Biala et al., 2009; Yu et al., 2009; Fang et al., 2011; Rezayof et al., 2011; Hashemizadeh et al., 2014
	↔ alcohol CPP	Pina and Cunningham, 2014
	↔ cocaine SA	Cossu et al., 2001; Lesscher et al., 2005; Caillé et al., 2007; Xi et al., 2008; Schindler et al., 2016
	↓ MDMA SA	Sala and Braida, 2005; Touriño et al., 2008
	↓ nicotine SA	Cohen et al., 2002; Shoaib, 2008; Simonnet et al., 2013; Schindler et al., 2016
	↓ heroin/morphine SA	Cossu et al., 2001; Navarro et al., 2001; Solinas et al., 2003
	↓ alcohol drinking	reviewed in Pava and Woodward (2012)
	↓ alcohol SA	Freedland et al., 2001; Navarro et al., 2001; Caillé et al., 2007
	↓ THC SA	Tanda et al., 2000; Schindler et al., 2016
	↓ cocaine seeking	De Vries et al., 2001; Xi et al., 2008; Orio et al., 2009; Ward et al., 2009; Vaughn et al., 2012; Jing et al., 2014; McReynolds et al., 2016; Schindler et al., 2016
	↓ nicotine seeking	Cohen et al., 2005; Kodas et al., 2007; Schindler et al., 2016
	↓ heroin seeking	Fattore et al., 2005; Caillé and Parsons, 2006; Alvarez-Jaimes et al., 2008
	↓ alcohol seeking	de Bruin et al., 2011
	↓ THC seeking	Justinova et al., 2008; Schindler et al., 2016
CB1 neutral antagonist	↔ cocaine SA	Schindler et al., 2016
	↓ nicotine SA	Gueye et al., 2016; Schindler et al., 2016
	↓ THC SA	Schindler et al., 2016
	↓ cocaine seeking	Schindler et al., 2016
	↓ nicotine seeking	Gueye et al., 2016; Schindler et al., 2016
	↓ THC seeking	Schindler et al., 2016
CB1 agonist	↑ nicotine SA	Gamaleddin et al., 2012
	↑ nicotine seeking	Gamaleddin et al., 2012
CB1 allosteric modulator	↓ cocaine seeking	Jing et al., 2014
	↓ methamphetamine seeking	Jing et al., 2014

The situation appears to be more complex for cocaine, as CB1 antagonists or knockout do not prevent self-administration of this drug (Cossu et al., 2001; Lesscher et al., 2005; Caillé et al., 2007; Xi et al., 2008). However, these antagonists reduce reinstatement of cocaine seeking produced by stress, drug-associated stimuli, or priming with the drug itself (De Vries et al., 2001; Ward et al., 2009; Vaughn et al., 2012; Jing et al., 2014; McReynolds et al., 2016), and also reduce cocaine seeking in a progressive ratio task and cocaine enhancement of intracranial self-stimulation (aka rewarding brain stimulation; Xi et al., 2008; Orio et al., 2009). “Impulsive” responding for cocaine in a delay-discounting task is also reduced by a CB1 antagonist (Hernandez et al., 2014). It is not fully clear if decreased intake is the result of one common eCB/CB1 action, or if several mechanisms are involved. Cocaine treatment mobilizes eCB production in the VTA, and thus suppresses GABAergic inhibition of DA neurons, resulting in enhanced DA release in the NAc (Wang et al., 2015). Inhibition of drug-induced increases in DA levels in the NAc is one consequence of CB1 antagonist treatment that could reduce the rewarding effects of drugs as well as drug intake (Cheer et al., 2007; Li et al., 2009; Wang et al., 2015). The DA-dampening effect of CB1 antagonists has been seen when CB1 antagonists are co-administered with several different drugs of abuse (Polissidis et al., 2014). However, other interactions with the eCB/CB1 system could alter drug reward and intake. Indeed, one recent study demonstrated that deletion of CB1 from forebrain GABAergic neurons (including striatal MSNs) enhances DA release (Martín-García et al., 2016), indicating that CB1 receptors at multiple synapses regulate the neurochemical effects of cocaine, and perhaps other abused drugs (Miller et al., 2007).

The ability of CB1 antagonists such as rimonabant to reduce the rewarding properties of several drugs of abuse and inhibit reinstatement of drug seeking following abstinence led to the idea that CB1 receptor blockade could be a viable therapeutic approach for treating drug use disorders (reviewed in Justinova et al., 2009). However, clinical evaluation of rimonabant, which is an inverse agonist of CB1 receptors, revealed substantial adverse effects including production of psychiatric symptoms (e.g., depression, anxiety) as well as gastrointestinal symptoms (Kaur et al., 2016). Interestingly, recent efforts have focused on the development of CB1 receptor neutral antagonists rather than inverse agonists (e.g., AM4113; Kirilly et al., 2012). Importantly, recent preclinical studies demonstrate that, like rimonabant, neutral antagonists of CB1 reduce self-administration of Δ^9^-THC and nicotine, and can also prevent cue-induced reinstatement of Δ^9^-THC, nicotine, and cocaine seeking (Gueye et al., 2016; Schindler et al., 2016). These results suggest that development of neutral antagonists could represent a renewed opportunity to therapeutically target CB1 receptors.

In addition to neutral antagonists, efforts have been made to develop allosteric modulators of CB1 receptors as an alternative to inverse agonists. For example, ORG27569 binds to an allosteric site on CB1 receptors and enhances agonist binding but reduces agonist efficacy, thereby acting as a functional negative allosteric modulator (Price et al., 2005; Picone and Kendall, 2015). Importantly, ORG27569 reduces both drug-priming and cue-induced reinstatement of cocaine and methamphetamine seeking behavior (Jing et al., 2014), suggesting that allosteric modulation provides an additional alternative strategy for drug development. Intriguingly, the steroid hormone precursor pregnenolone was recently identified as a putative endogenous negative allosteric modulator of CB1 receptors (Vallée et al., 2014). Accordingly, pregnenolone reduces behavioral and physiological effects of Δ^9^-THC administration. Additional studies will be necessary to determine if negative allosteric modulators of CB1 have improved safety and tolerability profiles when compared with inverse agonists.

Chronic exposure to several different drugs of abuse results in different molecular and functional changes in the eCB/CB1 system. Perhaps the easiest to understand are the effects of Δ^9^-THC and other CB1 agonists on CB1 expression and function. Injection of Δ^9^-THC in rodents once (Mato et al., 2004), or over several days (Mato et al., 2005; Nazzaro et al., 2012), results in loss of synaptic depression induced by eCBs and CB1 agonists. This loss of plasticity has been observed at glutamatergic synapses in dorsal and ventral striatum (Mato et al., 2005; Nazzaro et al., 2012; Hoffman and Lupica, 2013), as well as GABAergic synapses in hippocampus (Mato et al., 2004). Measurements of CB1 radioligand binding have provided mixed results, with some studies indicating that receptor numbers are diminished by this chronic drug exposure, while others report no change (Abood et al., 1993; Rodríguez De Fonseca et al., 1994; Romero et al., 1997; Mato et al., 2005). Measuring biochemical signals very proximal to CB1 activation using GTPγS binding supports the idea that receptor coupling to G proteins is compromised following chronic Δ^9^-THC treatment (Breivogel et al., 1999; Mato et al., 2005). This may signal internalization of receptors leading to alterations in G protein interaction. Until very recently it was difficult to measure CB1 internalization in presynaptic terminals, but a recent study using super resolution microscopy in hippocampal GABAergic terminals indicated that chronic Δ^9^-THC treatment does indeed produce internalization of CB1 (Dudok et al., 2015). Receptor localization returns to normal only several weeks after the end of drug exposure. It is also not clear if chronic Δ^9^-THC effects on CB1 are the consequence of direct ligand-receptor interactions, or occur secondarily to other molecular changes induced by the drug.

Use of cannabis-derived drugs have also been associated with decreased CB1 receptor availability in human ventral striatum, as determined with positron emission tomography using new CB1 ligands (Ceccarini et al., 2015). Other changes in human striatum have been noted in regular cannabis users, including decreased drug-induced DA release (Volkow et al., 2014). One report indicated that chronic cannabis drug use was associated with increased gray matter in young adult human NAc (Gilman et al., 2014), but a subsequent report indicated that most of this effect could be attributed to co-morbid alcohol use (Weiland et al., 2015). Thus, there is more work needed to elucidate cannabis drug effects on the human brain.

**Figure 2:**
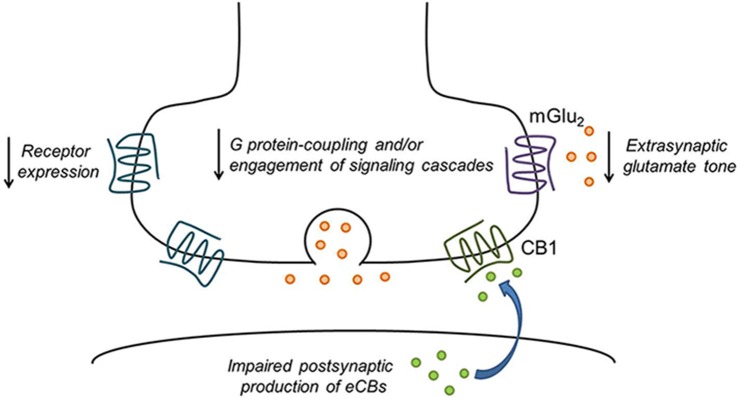
**Drug exposure disrupts presynaptic GPCR function.** A variety of mechanisms may be involved in drug-induced reductions in presynaptic inhibition of neurotransmitter release. These include reduced receptor expression or surface levels and impaired coupling to G proteins or activation of intracellular signaling cascades. CB1 receptor signaling may also be reduced due to deficits in postsynaptic endocannabinoid (eCB) production. In addition, reduced cystine/glutamate exchange leads to decreased tonic activation of perisynaptic mGlu_2/3_ in the nucleus accumbens (NAc). Finally, chronic drug exposure could cause long-term depression (LTD) of neurotransmitter release that occludes further synaptic modulation by GPCRs.

Among the non-cannabinoid drugs shown to alter eCB/CB1 function are alcohol, cocaine, nicotine and opiates. Acute alcohol inhibition of the firing of NAc neurons appears to involve an interaction with eCB/CB1 effects on afferents from the basolateral amydala (BLA; Perra et al., 2005). Interactions between acute alcohol exposure and eCB/cannabinoid actions have been observed in the BLA (Talani and Lovinger, 2015) and the central amgydala (CeA; Roberto et al., 2010). Chronic non-contingent alcohol exposure has been shown to eliminate eCB-dependent LTD at corticostriatal synapses in rodents (Xia et al., 2006; DePoy et al., 2013), and similar effects were observed following alcohol drinking (Adermark et al., 2011). Inhibition of GABAergic synapses by CB1 is also decreased following chronic ethanol exposure in both BLA and CeA (Varodayan et al., 2016a,b). A single *in vivo* exposure to cocaine also eliminates eCB-dependent LTD in the NAc (Fourgeaud et al., 2004), and effects of cocaine self-administration on glutamatergic synapses in the NAc shell involves an eCB-mediated mechanism (Ortinski et al., 2012). Following withdrawal after cocaine self-administration, the mGlu receptor/eCB-dependent LTD in the NAc is lost, and a different form of LTD develops that involves mGlu receptor-induced AMPA receptor trafficking (McCutcheon et al., 2011). Conversely, CB1 modulation of GABAergic transmission is sensitized following repeated cocaine exposure (Centonze et al., 2007). Reduced CB1 receptor expression has been observed in postmortem brain samples from human cocaine addicts (Álvaro-Bartolomé and García-Sevilla, 2013), and this was recapitulated in mice exposed to cocaine. Some studies report increased CB1 receptor expression levels despite reduced function, and this is likely due to the decreased eCB tone producing a compensatory upregulation of receptor levels (Blanco et al., 2014). A single *in vivo* exposure to the opiate drug oxycodone eliminates eCB-LTD in the dorsal striatum (Atwood et al., 2014a). It is clear that the effects of drugs of abuse on CB1 receptor function may be synapse-specific, drug-specific, and affect multiple aspects of the system including expression or function of enzymes that produce or degrade eCBs and the function of the CB1 receptor itself (Figure 2).

The effects of chronic exposure to cannabinoid drugs that produce addiction-related changes in behavior other than those discussed previously are not fully understood. An intriguing report of studies in mouse indicated that repeated injections of Δ^9^-THC leads animals to change their learned instrumental behavioral from goal-directed to habitual control (Nazzaro et al., 2012). This behavioral change is associated with the loss of striatal LTD discussed in the previous paragraph. Development of habits related to cannabis seeking and taking may well contribute to disorders associated with long-term heavy use of the drug. Determining the molecular and cellular mechanisms underlying habitual cannabis use will be an important subject of future research.

## Group II Metabotropic Glutamate Receptors

Glutamate is the major fast excitatory neurotransmitter in the CNS. It exerts its neurophysiological effects through both ionotropic and neuromodulatory metabotropic receptors. The mGlu receptors (mGlus) are a group of class C GPCRs comprised of eight subtypes that are divided into three subgroups based on sequence homology, G protein-coupling specificity, and ligand-binding profiles (for review see Niswender and Conn, 2010). The group I mGlu receptors (mGlu_1_ and mGlu_5_) couple to G_q_ family G proteins and are predominantly located postsynaptically, whereas the group II (mGlu_2_ and mGlu_3_) and group III (mGlu_4_, mGlu_6_, mGlu_7_ and mGlu_8_) mGlu receptors couple to G_i/o_ family G proteins and frequently act as presynaptic auto- and heteroreceptors. Although the group III mGlu receptors, particularly mGlu_7_, have received attention in the addiction field, the current review will focus on the preclinical evidence that group II mGlu receptors (mGlu_2/3_) modulate the actions of drugs of abuse and represent potential therapeutic targets for the treatment of drug use disorders. Group II mGlu receptors are widely expressed in brain regions relevant to drug-related behaviors. Although physiological actions of postsynaptic group II mGlu receptors have been identified (for example see Otani et al., 2002; Walker et al., 2015; Jin et al., 2016), they are best known for their ability to inhibit presynaptic neurotransmitter release at glutamatergic terminals. Group II mGlu receptor activation depresses glutamate release in critical regions such as the mPFC (Otani et al., 1999, 2002; Huang and Hsu, 2008; Walker et al., 2015), dorsal striatum (Lovinger and McCool, 1995; Kahn et al., 2001), NAc (Manzoni et al., 1997; Robbe et al., 2002a,b), central amygdala (Neugebauer et al., 2000), and BNST (Grueter and Winder, 2005). A variety of mechanisms might contribute to inhibition of neurotransmitter release by group II mGlu receptors, including inhibition of voltage-gated calcium channels (Anwyl, 1999; Robbe et al., 2002a; Kupferschmidt and Lovinger, 2015), interference with vesicle fusion and release machinery (Kupchik et al., 2008, 2011), and activation of presynaptic potassium channels (Anwyl, 1999).

Because group II mGlu receptors modulate glutamate release in key addiction-related brain circuits, it stands to reason that reduced expression and/or function of these receptors following drug exposure could contribute to dysregulated glutamate transmission. To test this hypothesis, a variety of anatomical and functional approaches have been used to assess the effects of drug exposure on mGlu_2/3_ expression and activity. Experiments evaluating drug- and withdrawal-induced changes in mGlu_2_ mRNA and protein levels have yielded somewhat conflicting results, and variables such as the drug of abuse, brain region, species, subject age and time point relative to drug exposure and withdrawal may contribute to such discrepancies. Multiple reports suggest that nicotine increases mGlu_2_ expression in the striatum during chronic exposure, but that receptor levels are normalized or decreased by abstinence (Counotte et al., 2011; Pistillo et al., 2016). No nicotine-induced changes in mGlu_2_ expression were observed in the PFC or midbrain (Pistillo et al., 2016). Cocaine exposure does not cause any change in mGlu_2_ or mGlu_3_ mRNA levels in cortical regions or the extended amygdala (Cannella et al., 2013), but increases mGlu_2/3_ density in the dorsal striatum of rhesus monkeys (Beveridge et al., 2011). In rats, alcohol dependance reduces mGlu_2_ mRNA levels in the mPFC (Meinhardt et al., 2013). Evaluation of mGlu_2_ and mGlu_3_ mRNA levels in postmortem samples from human alcoholics has revealed decreased *GRM2* levels in the anterior cingulate cortex (ACC; Meinhardt et al., 2013) and upregulation of *GRM3* transcript in the hippocampus (Enoch et al., 2014). Conversely, human cocaine abusers show lower *GRM3* transcript in the hippocampus (Enoch et al., 2014). In rats, methamphetamine self-administration followed by abstinence decreases mGlu_2_ protein levels in the PFC, dorsal striatum, and NAc (Schwendt et al., 2012). Intriguingly, extinction of methamphetamine self-administration reverses the downregulation of mGlu_2_ in striatal regions, whereas the effect persists in the case of abstinence. This finding suggests that active learning processes, and not the mere absence of drug exposure, can regulate plasticity of mGlu_2_ expression. Such observations of learning-induced plasticity in receptor expression provide potential neurobiological mechanisms for successful behavior-based addiction interventions.

Studies employing GTPγS binding, electrophysiological measures of synaptic transmission, and *in vivo* microdialysis to assess the impact of drugs of abuse on group II mGlu receptor function have yielded similarly varied results, and sometimes contrast with predictions based on changes in expression levels. For example, a history of nicotine exposure produces long-term decreases in mGlu_2/3_ function in the VTA, PFC, NAc, amydala, hippocampus and hypothalamus (Liechti et al., 2007; Counotte et al., 2011) despite a lack of evidence of widespread downregulation of mGlu_2/3_ expression (Pistillo et al., 2016). Repeated cocaine exposure (either investigator-administered or self-administered) impairs mGlu_2/3_-mediated depression of excitatory transmission in the PFC (Huang et al., 2007; Xie and Steketee, 2008; Kasanetz et al., 2013) and central amygdala (Neugebauer et al., 2000), whereas mGlu_2/3_-mediated LTD of excitatory transmission in the NAc remains intact following cocaine self-administration (Kasanetz et al., 2010). In conflict with these findings, Hao et al. (2010) reported that cocaine self-administration increases agonist-induced GTPγS binding in the medial PFC and central amygdala as well as the VTA, BNST, and hippocampus. Interestingly, in this study increases in receptor efficacy were only observed using a long access model of cocaine self-administration, indicating that the experimental paradigm may also contribute to seeming discrepancies between studies. In alcohol-exposed rats, no change in agonist-induced GTPγS binding was observed (Kufahl et al., 2011), whereas the ability of the same mGlu_2/3_ agonist to reduce extracellular glutamate levels *in vivo* was reduced (Meinhardt et al., 2013). Importantly, studies interrogating the effects of drug exposure on both expression and function suggest that increased expression does not necessarily correlate with functional readouts of receptor activity. For example, Xi et al. (2002) found that repeated cocaine exposure increases mGlu_2_ and mGlu_3_ protein levels in the PFC, but that agonist-stimulated GTPγS binding and efficacy for reducing extracellular glutamate levels were reduced. Such disparities between expression and function emphasize the importance of experimentally assessing receptor activity to clarify the functional effects of drug exposure.

Activation of group II mGlu receptors can modulate the neurochemical and behavioral effects of acute exposure to drugs of abuse, which commonly increase extracellular DA levels in both the dorsal striatum and NAc. Interestingly, cocaine exposure elicits higher levels of DA release in the NAc of mice lacking mGlu_2_ (Morishima et al., 2005). Conversely, group II mGlu receptor agonists attenuate DA release in the rat NAc following administration of amphetamine (Kim et al., 2005; Pehrson and Moghaddam, 2010). mGlu_2/3_ activation also reduces amphetamine-induced DA release in the rat dorsal striatum (Pehrson and Moghaddam, 2010) and cocaine-induced DA release in the caudate nucleus of squirrel monkeys (Bauzo et al., 2009). The mechanism for mGlu_2/3_ modulation of amphetamine-induced DA release is unclear, as a group II mGlu receptor agonist does not modulate DA release evoked by *in vivo* electrical stimulation of the VTA or by L-DOPA administration (Pehrson and Moghaddam, 2010). Interestingly, the effects of mGlu_2/3_ agonists on neurochemical responses to drug exposure are not limited to DA; the mGlu_2/3_ agonist MGS0028 also reduces the increase in PFC serotonin levels following methamphetamine administration (Ago et al., 2011). Importantly, one study demonstrated that mGlu_2/3_-mediated attenuation of nicotine-induced DA release in the NAc shell only occurs in rats with a history of nicotine exposure, and only when the rats are in a context previously associated with nicotine exposure (D’Souza et al., 2011). This finding provides intriguing evidence that prior drug exposure induces plasticity in the ability of group II mGlu receptors to modulate the effects of drugs on DA transmission.

Group II mGlu receptor modulation of the neurochemical effects of exposure to psychostimulants correlates with decreased locomotor responses to amphetamine (Table 3, Cartmell and Schoepp, 2000; Pehrson and Moghaddam, 2010; Hanna et al., 2013; Arndt et al., 2014), methamphetamine (Ago et al., 2011; Hiyoshi et al., 2014), and phencyclidine (PCP; Hanna et al., 2013). Activation of both striatal and PFC mGlu_2/3_ may be involved in the observed suppression of psychomotor effects of stimulants, as intrastriatal mGlu_2/3_ agonist administration reduces locomotor responses to amphetamine (Mao and Wang, 1999) and intra-PFC injection of the mGlu_2/3_ agonist APDC attenuates locomotor activation by cocaine (Xie and Steketee, 2009). Administration of the mGlu_2/3_-selective agonist LY379268 also blocks the acquisition and expression of behavioral sensitization to amphetamine (Kim and Vezina, 2002; Kim et al., 2005). On the other hand, locomotor sensitization to cocaine is enhanced in mGlu_2_ knockout mice (Morishima et al., 2005). The synaptic mechanisms of mGlu_2/3_-modation of neurochemical and behavioral responses to drugs of abuse remain unclear, and may not be mediated by heteroreceptor activity on the terminals of dopaminergic neurons (Pehrson and Moghaddam, 2010). Future work could elucidate specific circuit-level mechanisms by which presynaptic GPCRs such as mGlu_2/3_ modulate the pharmacodynamics of drugs of abuse.

**Table 3:** **Behavioral effects of drugs targeting mGlu_2/3_**.

Pharmacological manipulation	Behavioral effect	Reference(s)
mGlu_2/3_ agonist	↓ locomotor response to cocaine	Xie and Steketee, 2009
	↓ locomotor response to methamphetamine	Ago et al., 2011; Hiyoshi et al., 2014
	↓ locomotor response to amphetamine	Mao and Wang, 1999; Cartmell and Schoepp, 2000; Pehrson and Moghaddam, 2010; Hanna et al., 2013; Arndt et al., 2014
	↓ amphetamine sensitization	Kim and Vezina, 2002; Kim et al., 2005
	↓ locomotor response to PCP	Hanna et al., 2013
	↓ cocaine SA	Bauzo et al., 2009; Hao et al., 2010; Lu et al., 2012; Karkhanis et al., 2016
	↔ cocaine SA	Justinova et al., 2016
	↓ methamphetamine SA	Crawford et al., 2013
	↓ amphetamine SA	Kim et al., 2005
	↓ nicotine SA	Liechti et al., 2007; Justinova et al., 2016
	↓ alcohol SA	Sidhpura et al., 2010
	↓ cocaine seeking	Lu et al., 2007; Bauzo et al., 2009; Lu et al., 2012; Martin-Fardon and Weiss, 2012; Cannella et al., 2013; Justinova et al., 2016
	↓ methamphetamine seeking	Kufahl et al., 2013
	↓ nicotine seeking	Liechti et al., 2007; Justinova et al., 2016; Moro et al., 2016
	↓ heroin seeking	Bossert et al., 2004
	↓ alcohol seeking	Zhao et al., 2006; Sidhpura et al., 2010
mGlu_2_ PAM	↓ cocaine SA	Jin et al., 2010; Dhanya et al., 2011, 2014
	↓ nicotine SA	Justinova et al., 2015; Li et al., 2016
	↓ alcohol SA	Augier et al., 2016
	↓ cocaine seeking	Jin et al., 2010
	↓ methamphetamine seeking	Caprioli et al., 2015
	↓ nicotine seeking	Justinova et al., 2015; Li et al., 2016
	↓ alcohol seeking	Augier et al., 2016
mGlu_2/3_ antagonist	↑ alcohol SA	Zhou et al., 2013

Multiple lines of evidence support the idea that group II mGlu receptors regulate drug taking across a variety of paradigms and drugs of abuse. In rats, mGlu_2/3_ activation reduces motivation to self-administer cocaine as determined by a reduced breakpoint under a progressive ratio reinforcement schedule (Hao et al., 2010; Karkhanis et al., 2016). Studies evaluating self-administration of many drugs of abuse in rats have demonstrated that mGlu_2/3_ activation by LY379268 reduces intake of amphetamine (Kim et al., 2005), methamphetamine (Crawford et al., 2013), alcohol (Sidhpura et al., 2010), and nicotine (Liechti et al., 2007). Conversely, the group II mGlu receptor-preferring antagonist LY341495 increases alcohol self-administration in rats (Zhou et al., 2013). In squirrel monkeys, LY379268 has been shown to reduce cocaine self-administration (Bauzo et al., 2009). However, a more recent report demonstrated a reduction in nicotine self-administration by LY379268, but no effect on cocaine self-administration (Justinova et al., 2016). Although few studies have examined the circuitry by which mGlu_2/3_ activation reduces drug self-administration, experiments evaluating cocaine and nicotine self-administration have implicated both VTA and NAc receptor populations as potential mediators of reduced drug intake (Liechti et al., 2007; Lu et al., 2012).

Recent studies assessing the effects of genetic alteration of mGlu_2_ receptors provide further support for the notion that mGlu_2_ can regulate the reinforcing effects of repeated drug exposure. For example, genetic deletion of mGlu_2_ enhances both locomotor sensitization and CPP in response to repeated cocaine exposure (Morishima et al., 2005). In addition, mice lacking mGlu_2_ display increased alcohol consumption and preference in a two-bottle choice paradigm (Zhou et al., 2013). Interestingly, a naturally occurring mutation that introduces a stop codon in the gene for mGlu_2_ (*Grm2* * 407) was recently identified in multiple lines of rats that are commonly employed in alcohol studies. Selectively bred alcohol-preferring (P) rats, which consume large amounts of alcohol and display relapse-like behaviors, are homozygous for *Grm2* * 407. Intercrossing studies suggest that this allele is directly linked to increased alcohol consumption and preference in P rats (Zhou et al., 2013). Further investigation of lines of rats selectively bred for alcohol-related behaviors has also revealed an association between *Grm2* * 407 and alcohol consumption (Wood et al., 2016). Importantly, this mutation is also present in many commercially available rat lines; therefore, extreme care must be taken when choosing strains and sources of rats for studying drug seeking and taking, particularly in regards to evaluating the effects of mGlu_2_ on drug-related behaviors. Conversely, rats lacking mGlu_2_ can serve as valuable controls for the specificity of mGlu_2_-targeted drug effects (Augier et al., 2016; Wood et al., 2016).

Following extinction of drug self-administration, pharmacological activation of group II mGlu receptors can reduce reinstatement of drug-seeking behavior. Experiments in rats evaluating the effects of mGlu_2/3_ activation on reinstatement of drug seeking by cues previously associated with drug self-administration show that mGlu_2/3_ activation attenuates reinstated seeking of cocaine (Lu et al., 2007; Cannella et al., 2013), methamphetamine (Kufahl et al., 2013), alcohol (Zhao et al., 2006) and nicotine (Liechti et al., 2007; Moro et al., 2016). LY379268 also reduces context-induced reinstatement of heroin seeking (Bossert et al., 2004) and drug priming-induced reinstatement of methamphetamine seeking (Kufahl et al., 2013). Because stress is a significant factor contributing to relapse in human addictive disorders, mGlu_2/3_ activation has also been evaluated in models of stress-induced reinstatement of drug seeking. LY379268 blocks stress-induced reinstatement of cocaine (Martin-Fardon and Weiss, 2012) and alcohol seeking (Zhao et al., 2006; Sidhpura et al., 2010) in rats. Recent studies evaluating the effects of LY379268 on reinstatement of drug seeking in squirrel monkeys highlight the concept that activation of group II mGlu receptors may differentially reduce drug seeking depending on the specific drug of abuse and the type of reinstatement paradigm. Interestingly, LY379268 blocked both priming- and cue-induced reinstatement of nicotine seeking, but blocked cue-induced but not priming-induced reinstatement of cocaine seeking (Bauzo et al., 2009; Justinova et al., 2016). This finding may have intriguing implications for the therapeutic utility of group II mGlu receptor activation because efficacy in non-human primates could more closely predict human responses to drug treatments. On the whole, the most consistent finding is that mGlu_2/3_ activation could be useful for preventing relapse induced by environmental cues across a wide range of drugs of abuse.

Several studies have evaluated the potential contributions of mGlu_2/3_ receptors in various addiction-related circuits to inhibition of reinstated drug seeking. Numerous lines of evidence suggest that presynaptic mGlu_2/3_ receptors on excitatory terminals in the NAc play key roles. Producing an alcohol-dependent state in rats reduces mGlu_2/3_ expression and function in mPFC-NAc circuitry, and selectively restoring mGlu_2_ expression using a viral strategy reduces cue-induced reinstatement of alcohol seeking (Meinhardt et al., 2013). In addition, intra-NAc infusion of mGlu_2/3_ agonists reduces context-induced reinstatement of heroin seeking in rats (Bossert et al., 2004). Interestingly, the eugeroic drug modafinil, which has also been implicated as an anti-relapse drug (Soyka and Mutschler, 2016), reduces priming-induced reinstatement of cocaine seeking in rats by raising extracellular glutamate levels and indirectly activating group II mGlu receptors in the NAc core (Mahler et al., 2014). Further mechanistic insight into the role of NAc group II mGlu receptors in reinstatement of drug seeking comes from a series of studies evaluating the effects of repeated cocaine exposure on glutamate homeostasis. Cocaine decreases extrasynaptic glutamate levels in the NAc by decreasing the function of the glial cystine/glutamate exchange system (Baker et al., 2003). In turn, reduced tonic activation of presynaptic group II mGlu receptors disinhibits synaptic glutamate release, causing strengthened glutamatergic transmission at PFC-NAc synapses and disruption of several forms of synaptic plasticity (Moussawi et al., 2009). Treatment with N-acetylcysteine (NAC), which boosts cystine/glutamate exchange, corrects cocaine-induced adaptations in NAc glutamatergic transmission and prevents cue- and priming-induced reinstatement of cocaine seeking in an mGlu_2/3_-dependent manner (Moussawi et al., 2009, 2011). These studies provide insight into the involvement of group II mGlu receptors in drug-induced neuroadaptations as well as a potential mechanism by which mGlu_2/3_ could be targeted for therapeutic purposes.

Additional evidence implicates group II mGlu receptors in both the VTA and the CeA as targets for blocking reinstatement of drug seeking. Infusion of LY379268 into the rat VTA reduces context-induced reinstatement of heroin seeking (Bossert et al., 2004) and priming-induced resintatement of cocaine seeking (Lu et al., 2012). In addition, activation of group II mGlu receptors in the CeA prevents cue-induced reinstatement of cocaine seeking following prolonged extinction training (incubation of craving; Lu et al., 2007). It is possible that mGlu_2/3_ receptors (and particularly mGlu_2_) in various addiction-related circuits regulate drug seeking elicited by different stimuli. However, this hypothesis has not been exhaustively tested and may also vary between drugs of abuse.

It is worthwhile to consider that several studies have found that activation of group II mGlu receptors can also reduce instrumental responding for natural rewards (i.e., palatable foods) and/or inhibit reinstatement of extinguished food seeking in both rats and squirrel monkeys. However, this effect is typically less robust than reduction of drug self-administration and is observed with higher doses of agonist than the minimal effective dose required to reduce drug self-administration (Peters and Kalivas, 2006; Liechti et al., 2007; Jin et al., 2010; Kufahl et al., 2011, 2013; Lu et al., 2012; Justinova et al., 2016). Other studies have not observed a reduction in food self-administration following treatment with mGlu_2/3_ agonists (Baptista et al., 2004; Bossert et al., 2006; Zhao et al., 2006). A variety of factors could account for the observed impairment of food self-administration, including a general reduction in motivation, a lessening of the rewarding properties of the food reinforcer, generation of an aversive state (e.g., nausea) by the mGlu_2/3_ agonist, or mild sedation. These potential non-specific effects of mGlu_2/3_ activation could limit the clinical utility of mGlu_2/3_ agonists or mGlu_2_ positive allosteric modulators (PAMs) for the treatment of drug abuse in humans; however, the common finding that lower doses of agonist are required to reduce drug self-administration vs. food self-administration suggests that a sufficient therapeutic window may exist to avoid adverse effects. Moreover, clinical studies of the mGlu_2/3_ agonist prodrug LY2142003 in schizophrenic patients suggest that activation of mGlu_2/3_ is generally safe and well-tolerated (Patil et al., 2007).

## Challenges and Progress Towards Novel Pharmacotherapies for Addiction: The Example of mGlu_2_

Given the extensive evidence from preclinical models that activation of group II mGlu receptors can reduce seeking and taking of a variety of drugs of abuse, both academic groups and pharmaceutical companies have pursued drug discovery programs aimed at developing ligands that can be tested clinically to treat human drug use disorders. However, traditional approaches to developing drugs targeting these receptors have encountered a variety of challenges. For example, successful efforts to develop ligands targeting the orthosteric (glutamate) binding site of mGlu receptors have produced amino acid analogs, which often do not possess pharmacokinetic properties suitable for human dosing (Conn et al., 2014; however, see Mezler et al., 2010). Additionally, a common goal of drug discovery programs is to achieve high selectivity for a specific receptor subtype. This serves multiple purposes, including the creation of preclinical tools to validate specific targets for a given disorder. Moreover, subtype-selective drugs should be less prone to adverse effects mediated by off-target activity. Because the glutamate binding site is highly conserved between mGlu receptor subtypes, the development of highly selective ligands targeting this site has proven difficult. In recent years, discovery efforts have shifted to focus on allosteric modulators, which bind to a site on the receptor distinct from the orthosteric binding site and either enhance or inhibit the activity of the endogenous agonist.

PAMs potentiate the effects of endogenous agonists via multiple mechanisms that include enhancing the affinity of the receptor for the endogenous ligand and increasing efficiency of receptor coupling to downstream effectors (i.e., G proteins). Thus, PAMs of mGlu receptors can increase the potency of glutamate, as well as increase the maximal efficacy of receptor activation in the context of low receptor expression levels. Because allosteric binding sites are less conserved among GPCR families, many drug discovery campaigns have successfully created subtype-selective ligands (Conn et al., 2014). From a preclinical perspective, this success has permitted evaluation of specific mGlu receptor subtypes for treating drug use disorders. The development of several PAMs that are highly selective for mGlu_2_ and have properties suitable for *in vivo* testing has enabled the identification of mGlu_2_ as the major group II mGlu receptor subtype that could be pursued as a therapeutic strategy for reducing drug taking and seeking across a variety of classes of drugs. For example, the mGlu_2_ PAM BINA, as well as other novel mGlu_2_-selective PAMs, reduce cocaine self-administration in rats (Jin et al., 2010; Dhanya et al., 2011, 2014). Similarly, the recently developed mGlu_2_-selective PAM AZD8529 modestly reduces self-administration of alcohol in rats (Augier et al., 2016) and reduces nicotine self-administration in both rats and squirrel monkeys (Justinova et al., 2015; Li et al., 2016). mGlu_2_ has also been implicated as a therapeutic target for relapse, as mGlu_2_ PAMs block cue-induced reinstatement of cocaine (Jin et al., 2010), methamphetamine (Caprioli et al., 2015), nicotine (Li et al., 2016), and alcohol (Augier et al., 2016) seeking in rats as well as cue- and priming-induced reinstatement of nicotine seeking in squirrel monkeys (Justinova et al., 2015). Interestingly, AZD8529 failed to block stress-induced reinstatement of alcohol seeking, suggesting that the effect of mGlu_2_ PAMs on reinstatement of drug seeking may be specific to particular stimuli (Augier et al., 2016).

A common challenge associated with the use of traditional agonists to treat CNS disorders is that agonists persistently activate the receptor, which can lead to desensitization, internalization, and thus tolerance to the drug effect. In support of this concept, a study by Liechti et al. (2007) evaluating the effects of the mGlu_2/3_ agonist LY379268 on nicotine self-administration found an initial robust decrease in self-administration; however, over the course of 14 days of treatment, rats gradually returned to their previous levels of responding for nicotine, indicating tolerance to the effects of LY379268. Because PAMs enhance responses to glutamate derived from synaptic or other endogenous sources, and typically do not directly activate the receptor in the absence of the endogenous agonist, they are more likely to maintain normal spatial and temporal receptor activation patterns, and thus are less likely to promote tolerance. Recent studies evaluating repeated dosing of the mGlu_2_ PAM AZD8529 demonstrated that this PAM persistently suppressed nicotine self-administration in rats (Li et al., 2016) and squirrel monkeys (Justinova et al., 2015), providing substantial support for the hypothesis that PAMs may possess an advantage over agonists with regards to tolerance. In contrast, Li et al. (2016) found that the ability of another mGlu_2_-selective PAM, AZD8418, to reduce nicotine self-administration in rats was susceptible to tolerance. These findings highlight the fact that not all PAMs of a given receptor behave similarly *in vivo*, which must be taken into account when designing studies evaluating their therapeutic potential. Interestingly, divergent results such as those noted here could provide insight into the pharmacological properties that are necessary for maintained suppression of drug intake, and thus provide an opportunity to optimize drugs for clinical use with these profiles in mind.

One potential drawback of therapeutic approaches that rely on pharmacological enhancement of presynaptic receptor function stems from multiple lines of evidence that the function of presynaptic GPCRs is impaired following chronic drug exposure. Particularly in cases in which the apparent loss of auto- or heteroreceptor activity could be caused by downregulation of receptor expression, it is possible that pharmacological activation of the remaining receptor population would be insufficient to restore modulation of neurotransmitter release. This concept was postulated by Meinhardt et al. (2013), who reported decreased mGlu_2_ mRNA levels in the prefrontal cortex of alcohol-dependent rats (Meinhardt et al., 2013). In this study, lentiviral rescue of mGlu_2_ expression in infralimbic PFC-NAc circuitry prevented cue-induced reinstatement of alcohol-seeking; however, the ability of pharmacological agents to mimic this rescue was not reported. As discussed above, numerous studies have reported efficacy of both agonists and PAMs of mGlu_2_ to decrease taking and seeking of various drugs of abuse, lending support to the idea that despite adaptations in mGlu_2_ function caused by chronic drug exposure, pharmacological interventions remain a viable strategy for correcting drug-related behaviors.

Recent progress towards the development of clinically useful drugs targeting mGlu_2_ creates an exciting opportunity to test the hypothesis that enhancing mGlu_2_ function will decrease drug seeking and taking in humans. Two mGlu_2_ PAMs, AZD8529 (AstraZeneca) and JNJ-40411813 (Janssen Pharmaceuticals, Inc., in collaboration with Addex Therapeutics), have been evaluated in small clinical trials (Cross, 2013; Salih et al., 2015). To date, no major safety or tolerability concerns have been reported. A small trial assessed the ability of JNJ-40411813 to reduce cigarette craving and smoking in male smokers, and found a trend towards reduced craving but not a reduction of the number of cigarettes smoked (Salih et al., 2015). The U.S. National Institute on Drug Abuse is currently sponsoring a multi-site clinical trial to evaluate AZD8529 for smoking cessation in females (Clinicaltrails.gov identifier: NCT02401022). The results of this study will provide insight into the potential for translating preclinical evidence for the beneficial effects of mGlu_2_ PAMs into efficacy in human populations. Interestingly, clinical studies using NAC have shown a modest ability to promote abstinence from cocaine, cannabis, and nicotine (reviewed in McClure et al., 2014), providing additional, albeit indirect, support for the idea that increasing mGlu_2_ activity could reduce drug seeking in humans. Additional clinical trials examining the ability of drugs targeting mGlu_2_ to prevent relapse and reduce the use of a variety of drugs will be necessary to more thoroughly interrogate the specific human addictive behaviors that are most likely to be impacted by mGlu_2_ activation.

## Concluding Remarks

Substantial research using preclinical models of drug abuse has demonstrated key roles for presynaptic G_i/o_-coupled GPCRs as mediators of the effects of abused drugs, substrates for neuroadaptations that impact behavior, and promising therapeutic targets for reducing drug intake and preventing relapse. These receptors include the three examples discussed above; however, this list is by no means exhaustive. For example, mu opioid receptors are important mediators of the rewarding effects of opiates such as heroin, and also undergo adaptations in function in response to drug exposure (Atwood et al., 2014a). Naltrexone, one of the few currently approved drugs for treating opioid and alcohol dependance, targets these opioid receptors. In addition, other mGlu receptors such as mGlu_7_ have been the subject of substantial preclinical investigation and may provide alternative targets for future drug discovery efforts (Li et al., 2013; Mao et al., 2013). In addition, many mechanistic questions remain regarding the discrete synapses and circuits that are impacted by presynaptic GPCRs to modify drug intake and seeking. As the use of contemporary neuroscience techniques such as optogenetics and circuit-specific genetic manipulations become more widespread, enhanced understanding of how specific circuits control behaviors will provide opportunities to target GPCRs that modulate critical addiction circuitry to correct pathological drug use.

Advances in the ability to assess neuroadaptations in human patient populations will provide exciting opportunities to test the translational potential of the preclinical work highlighted here. For example, the increasing availability of PET ligands suitable for human testing will allow interrogation of drug-induced changes in receptor availability that could confirm preclinical observations of reduced presynaptic GPCR expression following repeated drug exposure without relying on the use of postmortem brain samples. In addition, methods such as magnetic resonance spectroscopy that can be used to study neurotransmitter dynamics in humans create the opportunity to assess the impact of long-term drug abuse on glutamate transmission and to test the ability of clinical drug candidates that target presynaptic GPCRs to modulate glutamate transmission in the addicted brain (Hillmer et al., 2015; Volkow et al., 2015). Ultimately, variables such as the abused drug(s) and history of drug taking will likely play a role in the efficacy of pharmacological interventions targeting presynaptic GPCRs. When designing clinical trials, it will be important to consider how the temporal relationship of the experimental intervention to drug taking (current or after short or prolonged abstinence) affects efficacy, and how time-dependent adaptations in receptor expression and function may impact the ability of novel drugs to reduce drug taking or seeking behaviors.

## Author Contributions

KAJ and DML both conceived of idea and plan for the manuscript, and both participated in manuscript writing and editing. KAJ created the figures and tables.

## Conflict of Interest Statement

The authors declare that the research was conducted in the absence of any commercial or financial relationships that could be construed as a potential conflict of interest.
